# Post-traumatic intraosseous sheath development of the extensor carpi radials brevis tendon: a case report

**DOI:** 10.1186/s13256-024-04932-2

**Published:** 2024-11-29

**Authors:** Seela Hinrichs, Rich Snellings, Ryan Scholte

**Affiliations:** 1 Northwestern University, 811 Emerson St., #622, Evanston, IL 60201 USA; 2 ImageCare Radiology, LLC, 57 Route 47, Suite 212, Hackettsown, NJ 07840 USA; 3 Emory University, 52 Mountainside Rd, Mendham, NJ 07945 USA

**Keywords:** Distal radius fracture, Post-traumatic growth deformity, Interosseous tendon course, Extensor carpi radialis brevis tendon, Inflammatory disorders

## Abstract

**Background:**

Complications from closed treatment in children are rare but can include growth disturbances and deformities. This case report presents a unique, post-traumatic growth deformity in the distal radius, where the extensor carpi radialis brevis tendon developed an interosseous course.

**Case presentation:**

A 49-year-old Caucasian male presented with chronic, dull pain in the radiodorsal aspect of the right wrist, worsened by weightbearing and terminal flexion and extension. The patient had a history of a distal radius fracture from a motor vehicle accident in adolescence, which was treated nonsurgically. Current X-rays showed a lucent process along the distal lateral radius without aggressive features. Magnetic resonance imaging revealed the extensor carpi radialis brevis tendon in an interosseous position within the distal dorsal lateral radius, with a longitudinal tear of the tendon. A noncontrast computed tomography scan confirmed a complete bone bridge along the tendon’s interosseous component, limiting potential surgical access. The patient, found to have a positive Antinuclear Antibody (ANA), was treated with prednisone and later with methotrexate and folic acid, which alleviated symptoms.

**Conclusions:**

This case highlights a rare post-traumatic deformity following a distal radius fracture, where the extensor carpi radialis brevis tendon formed an intraosseous sheath. It underscores the importance of considering unique anatomical changes when planning surgical interventions. This is the first reported case of an interosseous tendon sheath development around a tendon postdistal radius fracture, expanding the spectrum of known complications.

## Introduction

Distal radius fractures are a common fracture in children, adults, and the elderly. They represent one in six fractures in urgent care centers and emergency rooms [1]. Clinicians routinely see many variations of these fractures and their treatment typically depends on the age/function of the patient and the fracture type/displacement [2]. In children, closed methods are most often employed given the potential for remodeling at the distal radius due to the physis. Complications arising from closed treatment of pediatric distal radius fractures are not very common but include delated union, malunion, nonunion, joint stiffness, contractures, myositis ossificans, avascular necrosis, algodystrophy, osteomyelitis, secondary osteoarthritis, growth disturbance, or deformities [3, 4]. In this case report, we present a post-traumatic growth deformity in the distal radius where the patient developed an interosseous course of the extensor carpi radialis brevis tendon.

We believe this post-traumatic growth deformity in the distal radius is unique and has not been reported in the past.

### Case report

**Fig. 1:**
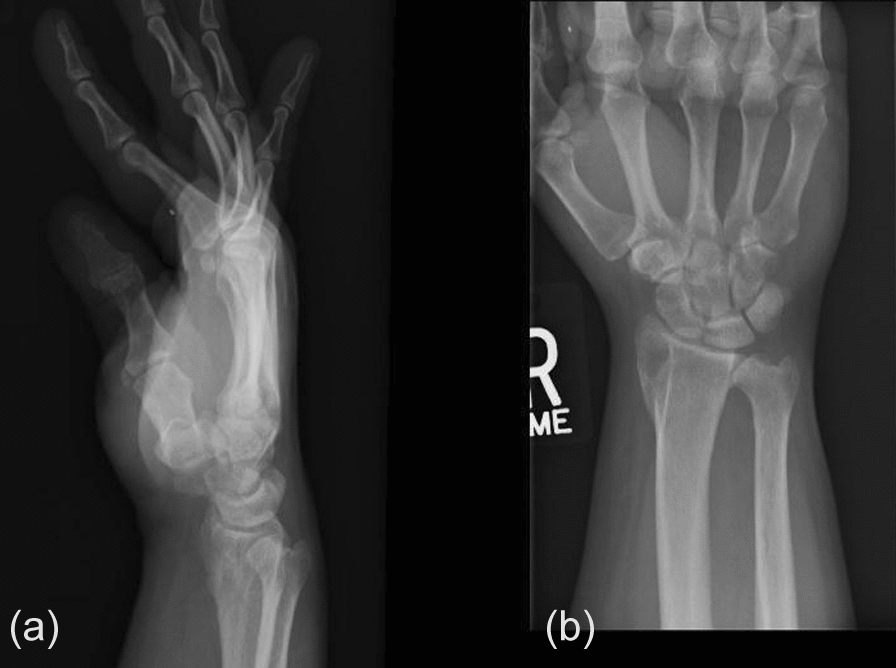
**a**, **b** Lucent lesion along the distal radial styloid

**Fig. 2:**
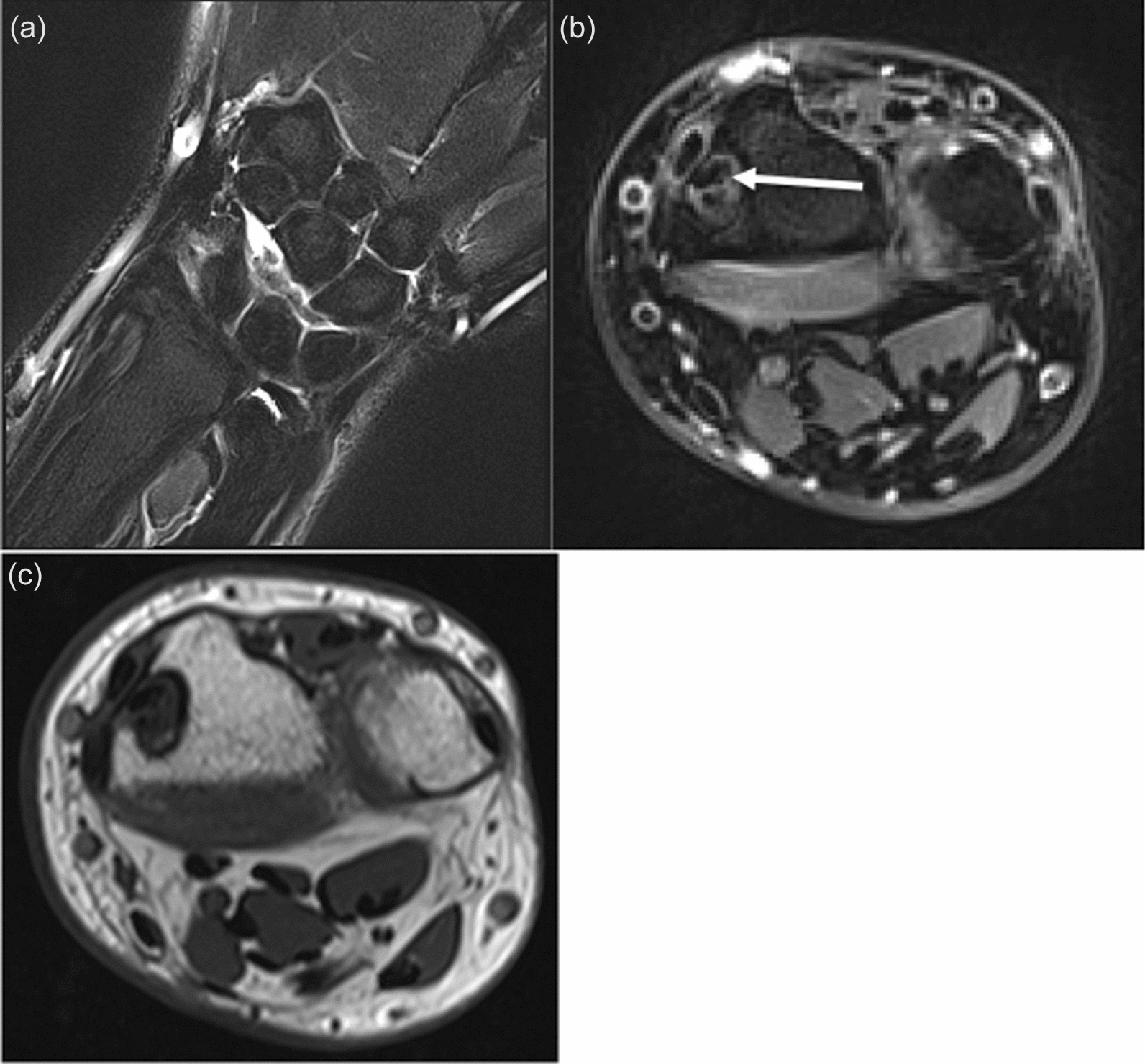
**a**, **b**, **c** Coronal proton density fat saturated image (**a**), axial proton density fat saturated image (**b**), and axial T1 image (**c**), all demonstrating a well-defined erosion with a well-corticated margin seen within the lateral side of the distal radius extending from the metadiaphysis to the epiphysis. The extensor carpi radials brevis tendon can be seen within this erosion. There is evidence of a longitudinal split tear within the extensor carpi radials brevis tendon with mild tendinosis, which is indicated by the white arrow on image b

**Fig. 3:**
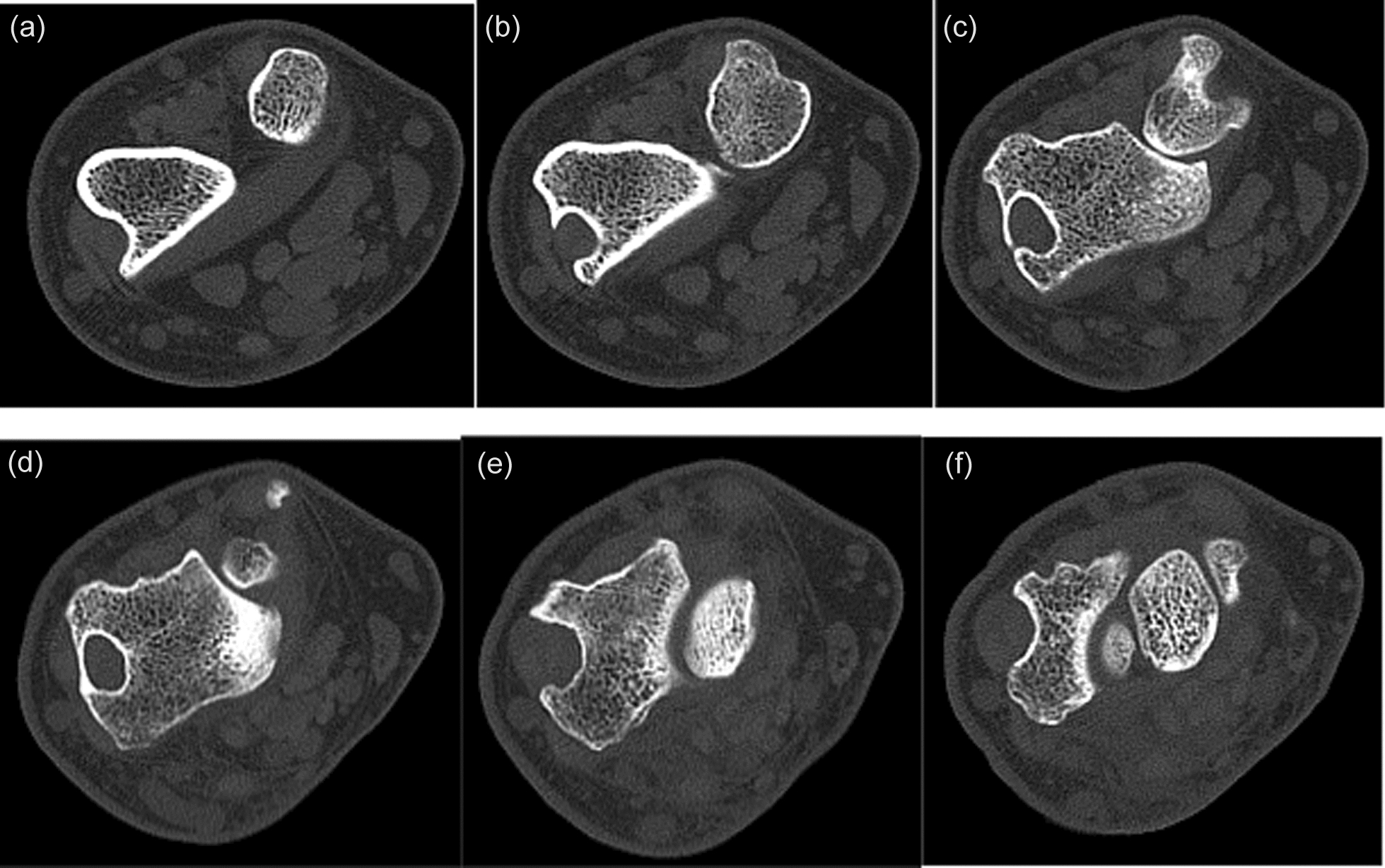
**a**, **b**, **c**, **d**, **e**, **f** Short-axis computed tomography slice through the distal radial ulnar joint demonstrating a lytic tract, which contains the extensor carpi radials brevis tendon both entering and exiting the tract. Note the complete bony bridge, which has formed along the course on Fig. 3**d**

Patient is a 49-year-old, right-hand-dominant Caucasian male who presented to the orthopedic clinic with insidious onset of dull, achy pain in the radiodorsal aspect of the right wrist. The pain worsened with weightbearing and terminal flexion and extension. The patient also described being injured in an motor vehicle accident (MVA) as a teenager and having his distal radius fracture treated with a cast; however, records from his prior injury were not available. X-rays from the office visit demonstrated a lucent process along the distal lateral radius. (Fig. 1a, b). There was no evidence of an internal matrix, soft tissue component, or other aggressive features. The patient was further evaluated with a pre- and postcontrast magnetic resonance imaging (MRI) of the wrist, which reflected the extensor carpi radials brevis tendon in an interosseous position in the distal dorsal lateral radius. (Fig. 2a, b and c). It was difficult to ascertain if this was located in an erosion or if there was a complete osseous tract within the distal radius. There was evidence of a longitudinal tear of the tendon within the tunnel. The tendon entered the distal radius dorsally and laterally extending along an interosseous course and subsequently exits the distal dorsal lateral radius near its articular surface with a proximal carpal row. The patient later had a noncontrast computed tomography (CT) scan to determine if he would be a viable candidate for the complete bone bridge across the superficial aspect of this tunnel. This potential solution would be less invasive than a surgical approach to the tendon for any anticipated repair. The CT showed a complete bridge of bone along the interosseous component of the tendon superficially. (Fig. 3a, b, c, d, and f). This, therefore, restricts any possible access to that tendon. The treating orthopedic surgeon recommended conservative treatment as it was determined that the potential risks associated with surgery to reposition the tendon outweighed the anticipated benefits. The patient was also evaluated by rheumatology and was found to have a positive Antinuclear Antibody (ANA). Prednisone alleviated his pain, but the pain returned after medication was discontinued. He was then prescribed methotrexate and folic acid, which improved his symptoms.

## Discussion

The typical clinical course of a distal radial fracture generally proceeds without significant complications, and they routinely heal without incident in response to nonsurgical management. This patient’s initial X-ray demonstrated an unusual lytic lesion with a sclerotic margin within the distal lateral radius. The patient provided a remote history of trauma. The CT and MRI findings demonstrated an intraosseous course of the extensor carpi radialis brevis tendon. The patient had no additional erosive changes or arthritic findings nor a history of arthritis, although he was found to be positive ANA.

Post-traumatic deformities are uncommon complications associated with distal radial fractures. The common deformities following distal radial fractures include the loss of the normal volar tilt of the articular surface in the sagittal plane, decreased ulnar inclination in the frontal plane and loss of length relative to the ulna [5]. When this patient suffered his initial injury in the MVA, he likely had a longitudinally oriented slightly displaced fracture. Our conclusion is that the intact tendon fell into that longitudinal grove produced by the displaced fracture. After the initial fracture healed around the intact tendon, the osseous sheath formed, as was seen on CT.

Inflammatory disorders can also alter tendon/bone healing [6]. Unfortunately, however, it is impossible to know if an inflammatory disorder impacted this particular patient during the time of healing. The patient showed no other clinical signs of an inflammatory condition other than a positive ANA.

## Conclusion

This case demonstrates an additional, unique, late post-traumatic complication of a distal radial fracture. This is the first-known reported case of an inter-osseous sheath development around a tendon.

## Data Availability

The authors declare that they had full access to all of the data in this study and the authors take complete responsibility for the integrity of the data and the accuracy of the data analysis.
